# Gut Microbiota as a Potential Modulator of Response to Biologic and Small-Molecule Therapies in Inflammatory Bowel Disease

**DOI:** 10.3390/ijms27219836

**Published:** 2026-11-03

**Authors:** Kacper Margul, Agnieszka Krawczyk

**Affiliations:** 1 Students’ Scientific Group of Microbiology, Faculty of Medicine, Jagiellonian University Medical College, 31-121 Krakow, Poland; kacper.margul@student.uj.edu.pl; 2 Department of Molecular Medical Microbiology, Chair of Microbiology, Faculty of Medicine, Jagiellonian University Medical College, 31-121 Krakow, Poland

**Keywords:** inflammatory bowel disease, gut microbiota, biologic therapies, small-molecule therapies, therapeutic response, drug–microbiota interactions

## Abstract

The therapeutic landscape of inflammatory bowel disease (IBD) has expanded substantially with the development of biologic and small-molecule therapies. Nevertheless, many patients fail to respond to these treatments or experience a secondary loss of response. The mechanisms underlying treatment failure remain incompletely understood. Given the ability of gut microorganisms to modify therapeutic compounds, sustain pro-inflammatory immune activation, alter intestinal barrier function and produce bioactive metabolites, the gut microbiota may represent an important modulator of therapeutic response. To summarize current evidence on the role of the gut microbiota in response to biologic and small-molecule therapies in IBD, with particular emphasis on potential mechanisms of drug–microbiota interactions and their implications for treatment response and non-response. Accumulating evidence suggests that the gut microbiota may influence therapeutic efficacy through direct modification of drugs and indirect effects mediated by host immunity, intestinal barrier function and microbial metabolites. Microbial signatures associated with response to individual biologic therapies have been identified, although findings remain heterogeneous. Emerging evidence also indicates that non-bacterial components of the gut ecosystem may contribute to treatment response. The gut microbiota represents a biologically plausible modulator of therapeutic response in IBD. Integration of microbiome data with host genetic and immunological features may help identify biomarkers capable of predicting response to individual therapeutic agents.

## 1. Introduction

Inflammatory bowel disease (IBD) comprises a group of chronic, nonspecific, idiopathic inflammatory disorders of the gastrointestinal tract, characterized by a relapsing-remitting clinical course [1,2,3]. Crohn’s disease (CD) is one of the two major forms of IBD, distinguished by segmental, asymmetric, and transmural inflammation that frequently involves the terminal ileum and colon and extends through the full thickness of the intestinal wall. In contrast, ulcerative colitis (UC) is defined by chronic inflammation confined to the mucosal layer of the colon, manifesting as immune cell infiltration into the mucosa, epithelial cell damage, and ulceration [2,3]. The disease burden of IBD is considerable: patients experience debilitating symptoms, including abdominal pain, severe diarrhea, bloody stools, tenesmus, weight loss, malnutrition, and fatigue, with complications ranging from colonic perforation and severe intestinal bleeding to an elevated risk of colitis-associated colorectal cancer [2,4,5,6]. This clinical impact is substantial as the disease markedly impairs patients’ quality of life and frequently necessitates long-term medical treatment, hospitalization, and surgical intervention. Despite major advances in medical therapy, approximately 10–20% of patients with UC ultimately require proctocolectomy because of medically refractory disease, highlighting the persistent unmet clinical need [2,4]. Similarly, a substantial proportion of patients with CD eventually require surgery, most commonly for stricturing or obstructive disease, abscesses, or fistulae, further highlighting the ongoing need for effective therapeutic strategies [7].

IBD has evolved into a growing global health challenge, with the Global Burden of Disease (GBD) 2021 study estimating approximately 3.8 million prevalent cases worldwide in 2021. While incidence has stabilized in most Western countries, it continues to rise sharply in newly industrialized regions, including Asia, Africa, and Latin America, with East Asia—particularly China—showing the steepest increase in age-standardized incidence among all global regions [8,9].

**Figure 1:**
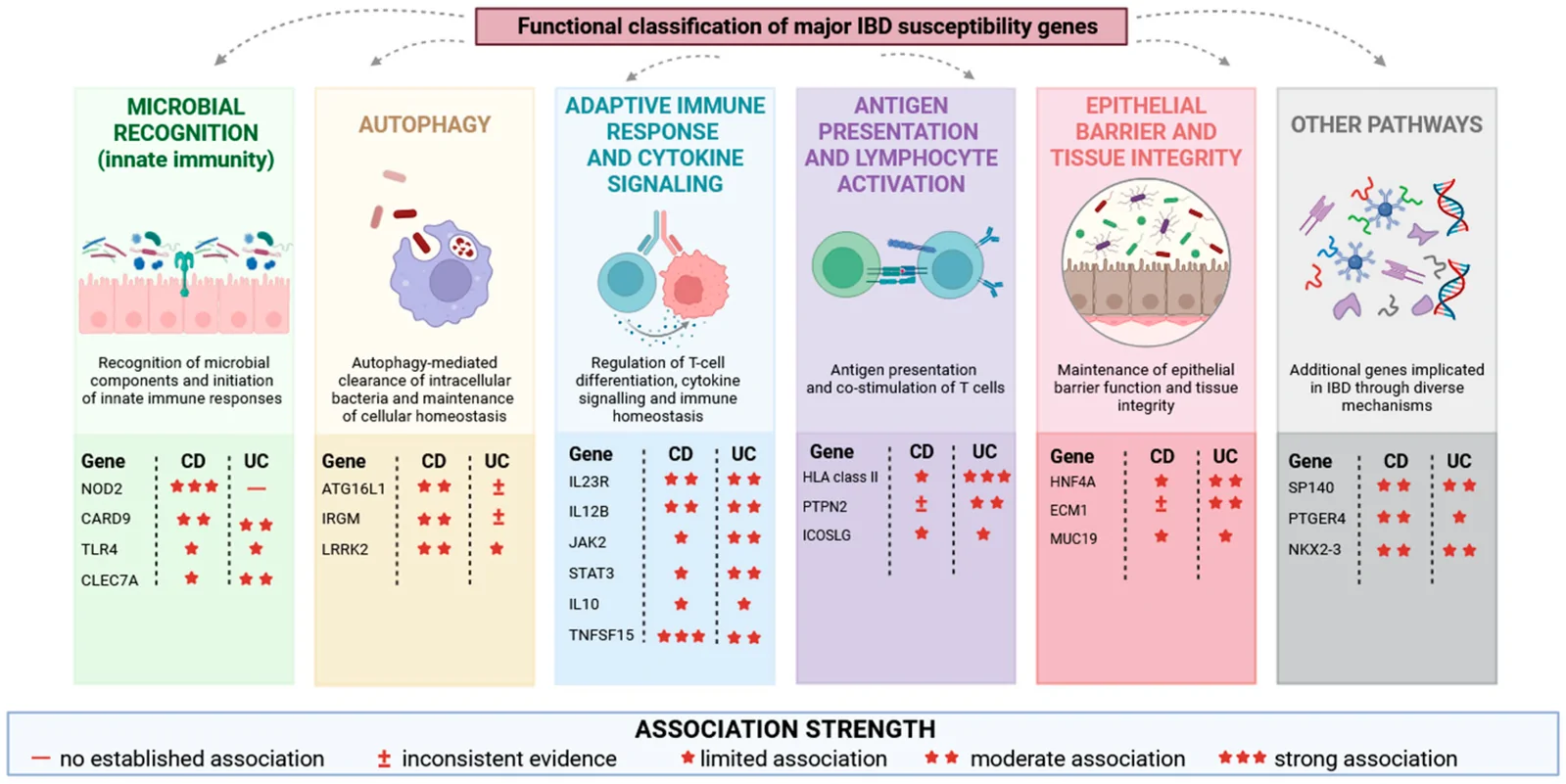
Functional classification of major inflammatory bowel disease susceptibility genes. Genes are grouped according to their predominant biological functions, with the strength of their associations with Crohn’s disease and ulcerative colitis indicated by the number of stars.

The etiology of IBD remains incompletely understood, but current evidence implicates an interplay of genetic, immunological, and environmental factors, whereby a genetically predisposed host mounts an inappropriate immune response to a triggering agent [10]; accumulating evidence points toward intestinal microorganisms as the principal candidate for this trigger [11,12,13,14,15]. Genome-wide profiling has identified over 200 susceptibility genes [16], most involved in lymphocyte activation, cytokine signaling, microbial recognition, and intestinal barrier function (Figure 1) [17].

The genetic background of IBD differs between CD and UC, with some susceptibility loci showing disease- or population-specific associations. NOD2/CARD15 is one of the best-established genetic risk factors for CD, particularly among individuals of European ancestry [16,18,19], whereas other genetic variants affecting epithelial barrier function, innate immune recognition, and adaptive immune regulation contribute to susceptibility to both CD and UC (Figure 1). Genetic susceptibility may converge with a dysregulated mucosal immune responses involving Th1- and Th17-associated pathways, including IL-12, IL-17, IL-22, IL-23, TNF-α, and IFN-γ [20,21]; NOD2, for example, encodes an intracellular pattern-recognition receptor that recognizes bacterial muramyl dipeptide to activate NF-κB signaling [16,19] and its dysfunction predisposes to opportunistic infection by otherwise non-pathogenic bacteria [22,23]. Alterations in such host–defense pathways may impair the ability to maintain immune tolerance toward the intestinal microbiota, thereby contributing to persistent mucosal inflammation. The therapeutic efficacy of cytokine-targeted biologic agents, particularly anti-TNF-α therapies, further corroborates the centrality of cytokine dysregulation in IBD pathogenesis [24].

Among environmental factors implicated in IBD—including smoking [25], psychological stress [26], and hygiene-related exposures [27,28]—the gut microbiota carries the most extensive evidential support [29,30]. Rather than a single causative pathogen [31,32,33,34], current models favor dysregulated immune tolerance toward commensal intestinal microorganisms in genetically susceptible individuals, with quantitative shifts in microbial composition contributing to inadequate immune regulation and sustained mucosal inflammation.

The gut microbiota may therefore represent an important link between host susceptibility, intestinal inflammation, and therapeutic response in IBD. Accumulating evidence suggests that alterations in microbial composition and function may influence the efficacy of biologic and small-molecule therapies, potentially contributing to the substantial inter-individual variability in treatment response. Understanding these microbiota–drug interactions may provide new insights into the mechanisms underlying treatment response and failure and may help identify microbiome-based biomarkers for more individualized therapeutic decision-making. Accordingly, this review aims to summarize current evidence on the role of the gut microbiota as a potential modulator of response to biologic and small-molecule therapies in IBD, encompassing alterations across the bacterial, fungal, viral and archaeal components of the gut ecosystem and the mechanisms linking these microbial communities to therapeutic efficacy and treatment failure.

## 2. Literature Search Strategy

We conducted a comprehensive literature search to identify and evaluate studies investigating the role of the gut microbiota in modulating responses to biologic and small-molecule therapies in inflammatory bowel disease. Searches were performed in PubMed for articles published up to August 2026. The search strategy combined terms related to IBD (e.g., “inflammatory bowel disease”, “Crohn’s disease”, “ulcerative colitis”), the gut microbiota and its individual components (e.g., “gut microbiota”, “gut microbiome” “dysbiosis” “bacteriobiome” “mycobiome”, “virome”, “archaeome” and “microbial metabolites”), and therapeutic response (e.g., “biologics”, “biological therapy” “anti-TNF”, “vedolizumab”, “ustekinumab”, “JAK inhibitors,” “treatment response”, “treatment failure”, and “non-response”).

We included original preclinical and clinical research articles, as well as relevant review articles and meta-analyses, to integrate mechanistic evidence with clinical findings on the role of the gut microbiota in modulating response to biologic and small-molecule therapies in IBD. Articles published in languages other than English were excluded unless an English translation was available. Given the narrative nature of this review, our aim was not only to summarize the available evidence but also to critically synthesize findings across studies, identify recurring patterns, and draw conclusions regarding potential mechanisms linking the gut microbiota to treatment response. In contrast to reviews focusing on specific microbial components or individual therapeutic approaches, this review integrates evidence on bacterial and non-bacterial components of the gut ecosystem, microbial metabolites, and their potential roles in modulating responses to both biologic and small-molecule therapies in IBD. The key findings and potential therapeutic implications are summarized in tables to facilitate comparison across studies.

## 3. IBD Therapies and Variability in Treatment Response

**Figure 2:**
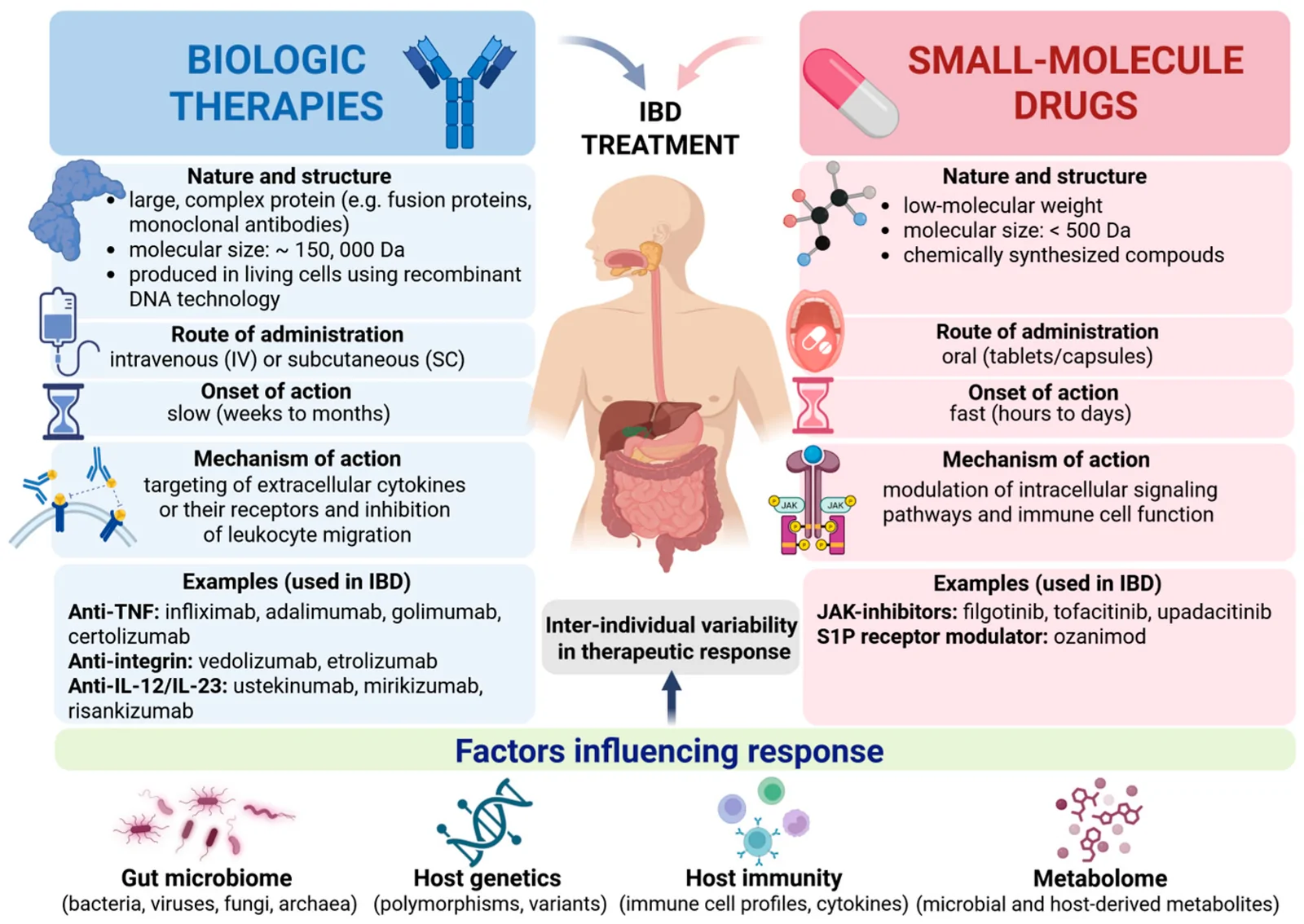
Biologic and small-molecule therapies for inflammatory bowel disease. Key differences in molecular structure, route of administration and mechanism of action are illustrated, together with representative therapeutic agents and factors potentially contributing to variability in treatment response.

Mesalazine constitutes the cornerstone of therapy, especially in UC, and is typically maintained lifelong [2,6]. In patients presenting with a more severe clinical course, corticosteroids and/or immunosuppressive agents (e.g., azathioprine, methotrexate) are introduced during flare-ups [2,35]. Nevertheless, a proportion of these patients develop steroid resistance or steroid dependence and fail to achieve remission with conventional treatment [1,2]. In this most severely affected subgroup, biological therapy serves as an essential therapeutic option [2,36]. The therapeutic armamentarium for IBD now spans two principal classes of advanced therapy: biologics and small molecules (Figure 2) [1,35].

Among biologic agents, anti-TNF antibodies such as infliximab and adalimumab act by antagonizing tumor necrosis factor-alpha, inducing T-cell apoptosis and interrupting the inflammatory cascade [2,35,37]. These agents are used to induce and maintain remission in patients with moderate-to-severe CD and UC. They can also promote mucosal healing and reduce hospitalization and surgical rates [2,37,38]. Nevertheless, up to 40% of patients exhibit primary non-response, and as many as 50% experience secondary loss of response over time [1,39]. Vedolizumab, an anti-integrin antibody targeting α4β7 integrin, instead achieves a gut-selective effect by blocking leukocyte migration into the gastrointestinal tract [1,2,37], avoiding systemic immunosuppression and offering an option following anti-TNF failure [1,38], albeit with a slower onset of action and comparable susceptibility to non-response [1,40]. Ustekinumab, targeting the shared p40 subunit of IL-12 and IL-23, diminishes the maturation and proliferation of pathogenic Th1 and Th17 cells and functions as an effective out-of-class alternative in patients refractory to anti-TNF therapy, though primary and secondary loss of response remain significant limitations across all three agents [2,4,38,40].

More recently, small-molecule therapies have emerged as a mechanistically distinct alternative: unlike biologics, which are large proteins targeting extracellular molecules, these orally administered, low-molecular-weight compounds are able to penetrate the cell membrane and act directly on intracellular signaling pathways. The JAK inhibitors filgotinib and upadacitinib block the Janus kinase (JAK-STAT) pathway, interrupting signal transduction downstream of multiple pro-inflammatory cytokines; upadacitinib is approved for moderate-to-severe CD, and both agents are used in moderate-to-severe UC refractory or intolerant to conventional or advanced therapies. Their oral administration and rapid onset of symptom relief, within one to seven days, are important advantages. However, their use is associated with potential side effects, including serious infection, major adverse cardiovascular events, venous thromboembolism, and malignancy [2,35,41].

Across both biologic and small-molecule classes, primary and secondary non-response remain pervasive and unresolved clinical challenges, motivating investigation into host-level factors—such as the gut microbiota—that may underlie this variability in treatment response [37,39,41].

Despite the growing therapeutic armamentarium available for IBD, the selection of treatment for an individual patient remains largely empirical, guided by standardized algorithms and decision trees rather than by patient-specific biological data—an approach that in practice amounts to a trial-and-error strategy [39]. This absence of a rational, individualized framework is closely linked to the lack of validated biomarkers capable of predicting a given patient’s response to a specific therapeutic agent, which continues to represent one of the major unmet needs in the field [36]. Such an empirical approach is not without consequence: exposing patients to successive, potentially ineffective treatment attempts carries the risk of unnecessary adverse effects, delayed disease control, and considerable healthcare costs [42]. Although numerous candidate biomarkers have been proposed over the past decade, none has yet been consistently validated or adopted into routine clinical practice, leaving the optimal selection of therapy for the individual patient an unresolved clinical challenge [3]. Overcoming this limitation is regarded as essential for the field to move beyond a one-size-fits-all treatment paradigm toward a precision medicine approach, in which therapeutic decisions are tailored to each patient’s individual immunological and microbial profile [43].

Among these host-level factors, the gut microbiota has emerged as a particularly compelling candidate, owing to its established bidirectional interaction with pharmacological therapy in IBD [37]. Accumulating evidence indicates that microbial composition and function can shape drug efficacy, toxicity, and clearance [37,39], that distinct taxonomic and functional signatures distinguish responders from non-responders to biologic therapy [42], and that multivariate models incorporating microbiome data outperform purely clinical predictors of remission [44].

## 4. Gut Microbiome Dysbiosis in IBD

A characteristic feature of IBD is dysbiosis of the bacteriome, alongside significant shifts in the populations of fungi, viruses, and archaea. Metagenomic analysis has shown that the number of microbial genes in the mucosa of IBD patients is reduced by 25% compared with healthy individuals [45]. Beyond this loss of gene richness, distinct dysbiotic signatures have been characterized at the community level. A prospective study found that 65.9% of patients with active IBD harbored the dysbiotic Bact2 enterotype—defined by low bacterial cell density and reduced diversity—compared with only 12.9% of healthy controls [46]. Consistent with this pattern, a significant decrease in the Firmicutes/Bacteroidetes (F/B) ratio has been reported in both CD and UC patients [47].

Beyond global shifts in community structure, IBD is characterized by the selective depletion of specific taxa with anti-inflammatory properties. *Faecalibacterium prausnitzii*, one of the most abundant commensals in the healthy gut, is markedly reduced in IBD. This species produces butyrate, which maintains Th17/Treg balance and suppresses the NF-κB pathway [45,47]. Similarly, *Roseburia hominis* and *Roseburia intestinalis* are significantly diminished, impairing the conversion of acetate to butyrate within the mucosal layer [48]. Non-toxigenic strains of *Bacteroides fragilis* (NTBF), which produce polysaccharide A (PSA)—a molecule known to promote the development of anti-inflammatory Treg cells—are likewise depleted in IBD patients [48]. This pattern extends to other taxa: reduced abundance of *Bifidobacterium* (particularly *B. longum* and *B. adolescentis*), *Lactobacillus*, and *Akkermansia muciniphila* has also been confirmed [45,49].

Alongside the depletion of protective commensals, IBD is characterized by the expansion of pathobionts—organisms with pro-inflammatory potential. One prominent example is the expansion of facultative anaerobes within the phylum *Proteobacteria*, particularly members of the family *Enterobacteriaceae* such as *Escherichia coli*. According to the “oxygen hypothesis”, inflammation increases luminal oxygen availability, creating conditions that favor facultative anaerobes over obligate anaerobic commensals [50]. *Ruminococcus gnavus* is also enriched in IBD, and may account for up to 69% of the microbiota in some cases of CD. This species produces the pro-inflammatory polysaccharide glucorhamnan, which stimulates TNF-α secretion by dendritic cells [47,49]. Adherent-invasive *E. coli* (AIEC) represents a further pathobiont implicated in disease pathogenesis: these strains adhere to and invade the intestinal epithelium via the CEACAM6 receptor, promoting IL-1β secretion and Th17 cell activation [48,51].

While bacterial dysbiosis has been the primary focus of IBD microbiome research, alterations in the fungal, viral, and archaeal fractions of the gut microbiota are increasingly recognized as contributing to disease pathogenesis.

Quantitative shifts in the gut mycobiome are well documented in IBD, most notably an increased Basidiomycota-to-Ascomycota ratio, a pattern captured by the so-called fungal dysbiosis index [52]. At the genus level, this shift is characterized by an expansion of *Candida* (including *C. albicans*, *C. tropicalis* and *C. glabrata*) alongside a corresponding decline in *Saccharomyces* [50,53]. These compositional changes have direct immunological consequences: *C. albicans* produces the toxin candidalysin, which damages macrophages and induces a Th17 response [52]. *Debaryomyces hansenii*, enriched in lesional tissue in CD, similarly contributes to disease by impairing epithelial wound healing [53].

Alterations in the intestinal virome, particularly its bacteriophage component, have also been implicated in IBD. An expansion of the order *Caudovirales* has been observed in IBD patients, correlating with reduced bacterial diversity. This association suggests that phages may indirectly drive bacterial dysbiosis rather than merely reflecting it [47]. Mechanistically, phages isolated from patients with active UC have been shown to induce increased IFN-γ secretion by immune cells via the TLR9 pathway, thereby exacerbating inflammation [48,51]. Notably, findings regarding overall virome diversity in IBD remain inconsistent: while Chen and Wang reported an overall increase in virome diversity, Wang et al. found that mucosal virome richness and evenness may in fact be reduced in UC relative to healthy controls [47,51]. This discrepancy may reflect differences in study populations, sampling sites (mucosal versus fecal) or sequencing methodology, and underscores the need for further standardized virome studies in IBD.

Within the archaeal fraction of the gut microbiota, two members of the genus *Methanobrevibacter* display divergent and potentially clinically relevant patterns of dysbiosis in IBD. *Methanobrevibacter smithii* is reduced in abundance in IBD patients with levels increasing during remission, positioning this species as a potential biomarker of disease activity [47]. In contrast, *Methanobrevibacter stadtmanae* is enriched approximately three-fold in IBD patients and has been shown to strongly stimulate the production of pro-inflammatory cytokines, including TNF-α and IL-1β, by dendritic cells [47,49].

Collectively, these findings indicate that IBD-associated dysbiosis extends well beyond the bacteriome, encompassing coordinated shifts across the fungal, viral, and archaeal domains of the gut ecosystem. Importantly, microbial alterations associated with IBD should not be interpreted as predictors of therapeutic response unless their predictive value has been specifically evaluated. Such alterations may reflect underlying disease activity or inflammation rather than an intrinsic capacity to predict treatment outcomes.

## 5. Microbial Correlates of Diseases Activity, Severity and Recurrence

Beyond describing static compositional differences between the IBD and healthy gut microbiota, an increasing body of evidence links specific taxa to the dynamic course of disease, including disease activity, phenotype and postoperative recurrence. These associations underscorethe potential value of microbial taxa as clinical biomarkers.

The prognostic significance of *F. prausnitzii* extends beyond its general depletion in IBD. Its abundance is markedly lower during active disease than during remission. In the original cohort study by Sokol et al., [54] reduced levels of this species in patients with CD were associated with endoscopic recurrence following surgical resection. More recently, low ileal *F. prausnitzii* abundance has been shown to discriminate the ileal from the colonic CD phenotype, with a sensitivity of 82.50% and an AUC > 0.772, suggesting a role as a complementary prognostic marker [51].

A comparable pattern of clinical relevance is observed for AIEC. In a model of postoperative recurrence, the prevalence of AIEC in the neo-terminal ileum six months after surgery was twice that observed in the surgical specimen. Furthermore, the presence of AIEC in surgical tissue predicted severe endoscopic recurrence (aOR 3.36; *p* = 0.017). *E. coli* abundance is also significantly higher in CD than in either UC or healthy controls (*p* < 0.001), and can distinguish colonic CD from extensive UC (AUC 0.793) [51]. Mechanistically, AIEC strains not only adhere and invadethe intestinal epithelium via CEACAM6 but can also survive and replicate within macrophages, further promoting mucosal inflammation [48].

Fungal taxa likewise show associations with disease activity. Elevated abundance of *Candida tropicalis* and *Candida albicans* is observed during active UC with clinical improvement paralleled by a reduction in fungal load. *C. tropicalis* additionally engages in interkingdom interactions with *E. coli* and *Serratia marcescens*, forming thicker biofilms than either organism achieves in monoculture, thereby amplifying dysbiosis [52]. Within the genus *Malassezia*, *M. sympodialis* abundance rises globally during disease flares [47], while *M. restricta* is particularly enriched in the mucosa of CD patients and exacerbates intestinal inflammation via the CARD9 signaling pathway, as demonstrated in murine models [48,51].

Several additional taxa show activity- or recurrence-associated dynamics. *Ruminococcus gnavus*, already noted for its enrichment in IBD, shows a positive correlation with clinical disease severity in CD [47,52]. *Fusobacterium* abundance in the mucosa and stool correlates with fecal calprotectin, a marker of intestinal inflammation, and has been linked to postoperative recurrence in CD patients [46]. Consistent with its role as a marker of clinical improvement, fecal *Methanobrevibacter smithii* load is significantly reduced during active IBD and rises during remission [47]. *Roseburia* abundance, in turn, is negatively correlated with UC activity [45].

Collectively, these findings indicate that IBD-associated dysbiosis is not merely a static compositional signature but a dynamic process with the depletion of specific protective taxa—most notably *F. prausnitzii*—and the persistence of pathobionts such as AIEC being associated with disease activity and recurrence. Beyond these compositional changes, the functional activity of the gut microbiota may represent an important link between microbial dysbiosis, intestinal inflammation, and therapeutic response.

## 6. Microbial Metabolites as Regulators of Intestinal Inflammation

Beyond compositional shifts in the microbiota itself, a growing body of evidence highlights the functional consequences of dysbiosis: the depletion of key microbial metabolites that regulate host immune homeostasis, foremost among them short-chain fatty acids, tryptophan derivatives and bile acid metabolites.

### 6.1. Short-Chain Fatty Acids (SCFAs)

SCFAs—principally acetate, propionate and butyrate—are generated through bacterial fermentation of dietary fiber and resistant starch, chiefly by members of the *Firmicutes* and *Bacteroidetes*. Butyrate and propionate act as histone deacetylase (HDAC) inhibitors, suppressing NF-κB nuclear translocation and thereby reducing pro-inflammatory cytokine production (TNF-α, IL-1β, IL-6) while promoting Treg differentiation via enhanced Foxp3 expression [55]. SCFAs additionally signal through G protein-coupled receptors, including GPR41 (FFAR3), GPR43 (FFAR2) and GPR109A. Acetate-mediated activation of GPR43 triggers NLRP3 inflammasome activity and IL-18 release, reinforcing epithelial barrier integrity, while butyrate signaling through GPR43 on Th1 cells promotes anti-inflammatory IL-10 secretion [47,55]. Butyrate oxidation in colonocytes also maintains luminal hypoxia under physiological conditions. As noted above, the loss of butyrate-producing taxa in IBD increases epithelial oxygenation, favoring expansion of facultative anaerobes such as *Enterobacteriaceae* [50]. Consistent with these mechanisms, metagenomic analyses have shown that 12% of metabolic pathways differ between IBD patients and healthy controls with genes involved in butyrate and propionate metabolism markedly underrepresented [47,48]. A further, butyrate-independent anti-inflammatory mechanism has also been described for *F. prausnitzii*: a 15 kDa protein isolated from this species directly suppresses NF-κB signaling [47,48].

### 6.2. Tryptophan Metabolites

Dietary tryptophan is metabolized by gut bacteria, including *Clostridium sporogenes* and *Peptostreptococcus* species, into indole derivatives such as indole, indole-3-acetic acid, tryptamine and skatole. These metabolites activate the aryl hydrocarbon receptor (AhR), stimulating IL-22 secretion by CD4+ T cells and innate lymphoid cells (ILCs). IL-22, in turn, induces antimicrobial peptide release and strengthens the intestinal barrier [50,55]. Indole-3-acrylic acid, produced by *Peptostreptococcus*, additionally upregulates mucin gene expression and activates the anti-inflammatory Nrf2 pathway [47]. Clinically, IBD patients show significantly reduced plasma tryptophan levels, which correlate negatively with disease activity alongside decreased activity of pathways converting tryptophan to AhR ligands [47,50].

### 6.3. Bile Acid Metabolites

Primary bile acids, such as cholic acid, synthesized in the liver, are converted by the gut microbiota into secondary bile acids, including deoxycholic acid (DCA) and lithocholic acid (LCA) [50]. IBD patients exhibit a fecal shift toward primary bile acid accumulation with a corresponding depletion of secondary bile acids [47]. Secondary bile acids, particularly LCA derivatives, regulate Treg homeostasis and Th17/Treg differentiation, directly modulating the balance between pro- and anti-inflammatory lineages [47,48]. These effects are mediated through the farnesoid X receptor (FXR) and GPBAR1/TGR5: FXR activation preserves intestinal barrier integrity and suppresses pro-inflammatory cytokine production, an effect confirmed in DSS-induced colitis models [56]. This conversion depends on *Clostridium leptum*, whose depletion in UC results in DCA and LCA deficiency and consequently heightened inflammation [47,48]. Collectively, these findings support a self-reinforcing “vicious cycle” model of IBD pathogenesis: the depletion of SCFA-producing taxa raises luminal oxygen tension, favoring pathobiont expansion, which further erodes the microbial capacity to generate protective metabolites, perpetuating both dysbiosis and inflammation.

## 7. Mechanisms Linking the Gut Microbiota with Response to Biologic and Small-Molecule Therapies

### 7.1. Microbiota-Driven Immune Activation and Excessive TNF-α Production

Building on the dysbiotic, pathobiont-enriched microbial landscape described above, accumulating evidence indicates that specific taxa actively drive mucosal inflammation by engaging pattern-recognition receptors—Toll-like receptors (TLRs) and NOD-like receptors—culminating in NF-κB activation and the overproduction of TNF-α, IL-6 and IL-1β. This axis is increasingly recognized not only as a driver of disease activity but as a potential determinant of therapeutic response, particularly to anti-TNF biologics [53,57].

Among the implicated taxa, *Fusobacterium nucleatum* has emerged as a particularly well-characterized driver of this pathway through two independently reported mechanisms. Lei et al. demonstrated that mucosal *F. nucleatum* colonization in UC upregulates nicotinamide phosphoribosyltransferase (NAMPT) activating the NAD^+^ salvage synthesis pathway. The resulting rise in NAD^+^ drives p38 MAPK signaling and a marked increase in TNF-α, IL-1β, IL-6, IL-8 and IL-18 secretion in serum and colonic tissue in a DSS-induced murine colitis model involving *F. nucleatum* colonization. Critically, pharmacological inhibition of this pathway—using the NAMPT inhibitor FK866 (10 mg/kg) or the p38 MAPK inhibitor SB203580 (5 mg/kg)—not only reduced tissue TNF-α and IL-6, but restored responsiveness to infliximab, providing direct mechanistic evidence in this model that microbiota-driven cytokine induction can contribute to anti-TNF resistance [58]. Separately, Liu et al. showed that the *F. nucleatum* virulence factors FadA and Fap2 activate the TLR4/MyD88/NF-κB signaling cascade recruiting immunosuppressive cell populations and generating a pro-inflammatory microenvironment. Fecal microbiota transplantation from infliximab non-responders (NR-FMT) reproduced this phenotype in mice, driving M1 macrophage expansion and activation of NF-κB, MAPK, and TNF signaling [57].

Complementary evidence from review literature implicates several additional pathobionts in this activation axis. *Ruminococcus gnavus* strain ATCC 29149, already noted for its enrichment in IBD, produces an inflammatory glucorhamnan polysaccharide that activates TLR4 on dendritic cells and induce TNF-α secretion as demonstrated in experiments using murine bone marrow-derived dendritic cells [6,56]. Adherent-invasive *E. coli*, and specifically the reference strain LF82, engages the epithelial and macrophage receptor CEACAM6 via the FimH adhesin, triggering inflammatory cytokine production independently of LPS. Beyond this direct effect, AIEC also suppresses autophagy through an NF-κB–miR-130a/miR-30c axis that downregulates ATG5 and ATG16L1 expression, permitting intracellular bacterial persistence and further amplifying inflammation [49,59]. More broadly, the overgrowth of LPS-releasing *Enterobacteriaceae* characteristic of IBD dysbiosis activates pattern-recognition receptors, sustaining NF-κB signaling and driving massive TNF-α, IL-6 and IL-1β secretion in what has been described as a self-perpetuating cycle of dysbiosis and inflammation [49]. Enterotoxigenic *Bacteroides fragilis* (ETBF) contributes a further mechanism via its BFT toxin, which activates NF-κB, MAPK and STAT3/Th17 signaling, inducing COX-2 and IL-8 expression and compromising epithelial barrier integrity [6]. Finally, fungal pathogen-associated molecular patterns—such as cell-wall β-glucans—are recognized by C-type lectin receptors (Dectin-1, Dectin-2, Mincle), TLR4, and the NLRP3 inflammasome, converging on the adaptor protein CARD9 and NF-κB to drive IL-1β, IL-6, IL-12 and IL-23 release and Th1/Th17 polarization [53].

The mechanistic findings described above raise the possibility that sustained microbiota-driven TNF-α production may contribute to persistent inflammation during anti-TNF therapy. However, whether this inflammatory activity can exceed the neutralizing capacity of administered anti-TNF antibodies, such as infliximab, remains uncertain.

Experimental evidence from Lei et al. demonstrated that inhibition of the *F. nucleatum*-associated NAMPT/p38 MAPK pathway restored infliximab responsiveness in a murine colitis model, supporting a role for microbiota-driven inflammatory signaling in experimental anti-TNF resistance. However, this finding does not establish that TNF-α production exceeds the neutralizing capacity of the administered antibody [58]. Clinical correlative data also suggest a relationship between inflammatory status and treatment outcome. Studies cited within the review by Chen et al., including Martinez-Borra et al. and Mateos et al., reported a negative correlation between baseline plasma/serum TNF-α concentration and infliximab response in CD, with patients exhibiting very high baseline TNF-α levels achieving response less frequently (notably, elevated TNF-α instead favored response to ustekinumab) [60]. Similarly, Franzin et al. reported that non-responders to anti-TNF therapy presented with higher baseline levels of pro-inflammatory mediators—TNF-α, IL-6, IL-1β, IL-17a and IFN-γ—in serum and mucosal tissue, while higher infliximab trough levels and mucosal healing were associated with greater microbiota diversity and higher *F. prausnitzii* abundance [37]. These findings suggest a possible association between inflammatory activity and treatment response, but they do not demonstrate that microbiota-driven TNF-α production overwhelms antibody-mediated neutralization.

This picture is complicated, however, by contradictory findings. In a prospective cohort study, Lee et al. found that baseline serum concentrations of TNF, IL-12, IL-22 and IL-23 were not predictive of clinical or endoscopic remission at weeks 14 or 52 following anti-cytokine (anti-TNF/anti-IL-12/23) or anti-integrin therapy, indicating that baseline cytokine concentrations alone may be insufficient to predict treatment outcomes in the populations studied [61].

Overall, the available evidence suggests that anti-TNF resistance may reflect multiple interacting processes, including microbiota-driven inflammatory activation, epithelial metabolic dysregulation, and impaired barrier integrity. However, no study reviewed here directly demonstrates antibody stoichiometric saturation, whereby cytokine production exceeds the neutralizing capacity of administered antibodies. This proposed mechanism should therefore be regarded as a hypothesis rather than an established explanation for treatment failure. Although circulating anti-TNF concentrations may exceed circulating TNF concentrations, this does not establish that sufficient drug is available to neutralize TNF within inflamed intestinal tissue. Anti-TNF resistance is likely multifactorial, involving pharmacokinetic, immunogenic, and TNF-independent inflammatory mechanisms. More broadly, mechanistic evidence linking microbiota-driven inflammation to treatment resistance should be distinguished from evidence supporting the use of microbial features as pretreatment predictors of clinical response. Although inhibition of *F. nucleatum*-associated inflammatory signaling restored infliximab responsiveness in a murine model, whether baseline *F. nucleatum* abundance predicts anti-TNF response in patients with IBD remains to be established in prospective clinical studies.

### 7.2. Microbial Metabolism and Modification of Therapeutic Agents

A second, mechanistically distinct axis linking the gut microbiota to therapeutic outcome operates not through immune activation but through direct biochemical interaction with the drug molecule itself: bacterial enzymes can activate prodrugs, inactivate active compounds or structurally modify therapeutic agents, thereby altering their bioavailability and efficacy (Table 1) [37,62].

This principle is most clearly established for aminosalicylates. Sulfasalazine, olsalazine and balsalazide are administered as prodrugs whose azo bond must be cleaved by bacterial azoreductases—expressed by *Clostridium species* (*C. clostridiiforme*, *C. nexile*, *C. paraputrificum*, *C. perfringens*), *Eubacterium hadrum*, *Staphylococcus aureus*, *Escherichia coli*, *Klebsiella aerogenes*, *Pseudomonadaceae*, *Bacteroides*, *Lactobacillus acidophilus*, *Bifidobacterium lactis* and *Streptococcus salivarius*—to release active 5-aminosalicylic acid (5-ASA) in the colon; in germ-free or antibiotic-treated animals, sulfasalazine is excreted unchanged [37]. Once liberated, however, 5-ASA is itself subject to bacterial inactivation: N-acetyltransferases (NAT), expressed by genera including *Citrobacter*, *Klebsiella*, *Serratia*, *Shigella*, *Vibrio*, *Pseudomonas aeruginosa*, *Bacteroides*, *Clostridium* and *Lactobacillus*, acetylate 5-ASA to the inactive metabolite N-acetyl-5-ASA. High bacterial acetyltransferase activity has been linked to an increased risk of 5-ASA treatment failure and subsequent escalation to corticosteroid therapy [37,62].

A comparable dual pattern of bioactivation and inactivation governs the thiopurines (azathioprine, 6-mercaptopurine, 6-thioguanine). Nucleotide synthesis enzymes present in gut bacteria—HPRT/HGPRT, IMPDH and GMPS, identified in *E. coli* DH5α, *Enterococcus faecalis*, *Bacteroides thetaiotaomicron*, *B. fragilis* and *B. vulgatus*—convert these agents into active thioguanine nucleotides (TGN) with glutathione S-transferases in *Proteobacteria* further supporting the conversion of azathioprine to 6-mercaptopurine. Conversely, bacterial orthologs of human thiopurine methyltransferase (TPMT), described in *Pseudomonas syringae*, *P. aeruginosa* and *P. fluorescens*, methylate thiopurines and block their conversion to TGN, while bacterial xanthine oxidase in *E. coli* generates inactive thiopurine metabolites [37,62].

Glucocorticoids undergo both degradation and structural transformation by the gut microbiota. Bacterial glycosidases, sulfatases and reductases degrade second-generation corticosteroids released locally in the gut, such as budesonide and beclomethasone dipropionate. Incubation with human fecal microbiota degrades prednisolone within 3 h, budesonide within 7 h and prednisone within 96 h [37]. Beyond simple degradation, *Gordonibacter pamelaeae* and *Eggerthella lenta* perform 21-dehydroxylation converting glucocorticoids into progestogens and neurosteroids and thereby altering their original immunomodulatory activity [62].

Methotrexate is similarly subject to bacterial inactivation: carboxypeptidases hydrolyze the drug to 4-amino-4-deoxy-N10-methylpteroic acid (APA) [37], while glutamate carboxypeptidase II—identified in *E. coli*, *Prevotellaceae* and *Anaeroplasmataceae*—together with PGH and CPDG2 enzymes in *Lactobacillus casei* and *Pseudomonas* convert methotrexate to the inactive metabolite DAMPA [62].

Among small-molecule agents, susceptibility to microbial degradation varies markedly by drug class. The S1P receptor modulator ozanimod shows pronounced sensitivity to enzymatic degradation by the gut microbiota, with no more than 40% of the active compound remaining after 24 h of incubation. This finding was observed in experiments using fecal microbiota from six healthy donors, although the extent of ozanimod depletion varied between individual donors. After 24 h, ozanimod-derived metabolites accounted for 5–13% of the incubation mixture. By contrast, the JAK inhibitors—tofacitinib, baricitinib, and filgotinib remained largely intact under the same conditions with mean residual drug levels of 85%, 83%, and 85%, respectively, after 24 h of incubation [62,73]. These findings suggest that microbial metabolism may be relevant for the disposition of specific small-molecule therapies. However, the study was conducted in vitro, and it remains unclear whether the observed degradation occurs to a similar extent in patients with IBD or meaningfully alters drug exposure and therapeutic efficacy in vivo. More broadly, evidence on microbiota-mediated metabolism of small-molecule IBD therapies remains limited, highlighting the need for further studies to characterize microbial transformation pathways and establish their pharmacokinetic and clinical significance. Beyond drug degradation, the authors also investigated the effects of these agents on the gut microbial community. Their findings indicated that ozanimod altered microbial growth and metabolic profiles, whereas the tested JAK inhibitors had comparatively limited effects on the microbiota under the experimental conditions. This suggests that interactions between small-molecule therapies and the gut microbiome may be bidirectional, involving both microbial transformation of the drug and drug-induced changes in the microbial ecosystem [73]. However, these observations are based on in vitro experiments using microbiota from healthy donors, and their relevance to the intestinal environment and treatment outcomes in patients with IBD remains to be established.

Unlike the small-molecule agents discussed above, biologic therapies are not generally considered susceptible to the same microbial metabolic transformations because of their protein-based structure, high molecular weight, and parenteral administration. Nanni et al. described no evidence of direct bacterial metabolism of biologic agents in the context they reviewed [62]. The microbiota nonetheless appears to influence biologic drug exposure and immunogenicity indirectly through two distinct mechanisms. First, depletion of butyrate-producing, SCFA-generating bacteria compromises intestinal barrier integrity, promoting the leakage of therapeutic antibodies such as vedolizumab into the intestinal lumen and accelerating drug clearance [60]. Second, the microbiota appears to modulate the development of anti-drug antibodies (ADA): germ-free mice fail to generate ADA against infliximab, whereas microbiota exposure—and antibiotic-induced alterations to it—differentially affects ADA risk [60]. A potential direct proteolytic mechanism should also be distinguished from microbial metabolic transformation. The IgG-degrading enzyme IdeS, produced by *Streptococcus pyogenes*, has been shown to cleave infliximab in vitro, reducing its binding to complement factor C1q and the FcγRIIIA receptor and impairing associated effector functions (75–78). However, these findings do not establish that IdeS-mediated cleavage occurs at a clinically meaningful level in the intestinal environment of patients with IBD or that it contributes to treatment failure in vivo. Thus, although direct proteolytic cleavage of biologics is experimentally plausible, its contribution to clinical response in IBD remains insufficiently established. The current evidence supports a role for indirect microbiota-mediated pathways, while the clinical relevance of direct bacteria-derived proteolysis requires further investigation.

Importantly, the reviewed literature does not support the conclusion that direct enzymatic degradation of biologic molecules by intestinal bacterial proteases is an established mechanism in IBD. Although Han et al. note generally elevated proteolytic activity within the gut microbiota of UC patients [49], no study identified here provides evidence that bacterial proteases directly cleave or degrade anti-TNF, anti-integrin or anti-IL-12/23 antibodies or that such activity compromises their intestinal stability. The hypothesis that biologic agents are subject to direct enzymatic breakdown by gut bacteria therefore remains insufficiently established in the clinical context of IBD. The microbiota’s influence on this drug class appears to operate primarily through indirect pathways—barrier-mediated drug loss and modulation of host immunogenicity although the clinical relevance of direct bacteria-derived proteolysis requires further investigation [62].

Importantly, findings from bacterial cultures and fecal incubation experiments should be interpreted cautiously, as they do not necessarily reflect the extent or clinical consequences of microbial drug metabolism in vivo. The relevance of these processes depends on drug-specific pharmacokinetic properties, including the route of administration, intestinal exposure, absorption, and the location and duration of contact between the drug and the microbiota. In addition, the detection of a microbial transformation product does not establish whether the resulting metabolite retains pharmacological activity, is inactive, or has potentially different biological effects. For example, the degradation of a compound under in vitro conditions does not, by itself, demonstrate a clinically meaningful reduction in drug exposure or therapeutic efficacy in patients with IBD. Therefore, these findings should be considered mechanistic evidence, and their clinical significance requires validation in appropriate in vivo studies and, where possible, clinical cohorts.

### 7.3. Microbiota-Derived Metabolites Affecting Drug Response

The mechanisms described above—direct immune activation and drug biotransformation—are complemented by a third, more indirect pathway: microbial metabolites act on the host’s immune system and intestinal barrier, thereby modulating therapeutic response without directly contacting the drug itself (Table 2). As introduced earlier in this review, short-chain fatty acids, indole derivatives and secondary bile acids regulate immune receptor expression, Treg induction and epithelial barrier integrity. This section focuses specifically on evidence linking these metabolites to clinical outcomes on biologic and small-molecule therapy.

#### 7.3.1. Primary and Secondary Bile Acids

Bacterially modified bile acids have emerged as particularly direct determinants of biologic drug efficacy. Han et al. demonstrated, in a humanized-immune-system mouse model of TNBS-induced colitis, complemented by fecal microbiota transplantation, targeted metabolomics and single-cell RNA sequencing, that the secondary bile acid lithocholic acid acts as a ligand for TGR5 and FXR on intestinal macrophages. Activation of this TGR5/FXR–NF-κB axis suppresses p65/IκBα nuclear translocation, lowering pro-inflammatory markers such as iNOS and TNF-α while upregulating Arg-1, thereby reprogramming macrophages toward a pro-resolutive M2 phenotype. These reprogrammed macrophages restore epithelial tight-junction protein expression (ZO-1, Occludin), reduce disease activity index and serum TNF-α, and directly enhance vedolizumab efficacy in mouse model of CD. A combined baseline profile of microbiota and secondary bile acid metabolites predicted vedolizumab remission with an AUC of 0.917 [56].

A related mechanism has been described in the context of infliximab responseLiu et al. used MIMOSA2 metabolic modeling to implicate *Bifidobacterium catenulatum* DSM 16992 in taurochenodeoxycholic acid (TCDCA)-associated metabolic activity, however, direct production of TCDCA by this species was not established experimentally.

TCDCA is a taurine-conjugated primary bile acid that has been associated with regulatory T-cell (Treg) expansion and reduced M1 macrophage polarization and reduced neutrophil infiltration. Infliximab non-responders exhibited lower fecal TCDCA levels and reduced *Bifidobacterium* abundance. Furthermore, fecal microbiota transplantation from these non-responders exacerbated colitis in mice and was accompanied by reduced Treg frequencies, increased M1 macrophage polarization, and altered bile acid metabolism, including lower TCDCA levels. These findings support a link between microbiota-associated bile acid metabolism, immune dysregulation, and infliximab non-response, while the specific contribution of *B. catenulatum* to TCDCA production remains to be confirmed experimentally [57].

At the pathway level, Lee et al., in the prospective PRISM cohort, showed that conversion of primary to secondary bile acids depends on the bacterial bai operon (encoding the 7α/β-dehydroxylation enzymes BaiB, BaiCD, BaiE, and BaiH), carried predominantly by members of the order Clostridiales. Patients harboring a functional *bai* operon (*bai^+^*: 60.2% of the cohort) achieved significantly higher remission rates at week 14 on anti-cytokine biologics (anti-TNF/anti-IL-12/23) compared with anti-integrin therapy (OR 3.52, 95% CI 1.30–10.1, *p* = 0.02) and elevated serum secondary bile acids—including glycolithocholic acid (*p* = 0.04) and ursodeoxycholic acid (*p* = 0.03)—correlated negatively with fecal calprotectin [61]. This pattern is corroborated by Ding et al., who reported that anti-TNF non-responders exhibit elevated sulfated and glycine-conjugated primary bile acids, whereas high deoxycholic acid (DCA) levels favor remission, yielding a predictive model with an AUC of 0.81 [60]. The predictive significance of bile acid-related microbial features depends on the study design and the timing of sample collection. Baseline microbial and metabolic profiles associated with subsequent treatment outcomes may have value for pretreatment stratification, whereas differences observed between responders and non-responders without clearly established temporal relationships may also reflect disease activity or treatment-associated changes.

#### 7.3.2. Short-Chain Fatty Acids

Beyond their general immunomodulatory role outlined earlier—GPR41/43/109A signaling, HDAC inhibition and Treg induction—SCFAs, and butyrate specifically, show direct associations with therapeutic outcome. As noted in the discussion of biologic drug pharmacokinetics above, enrichment of butyrate-synthesis pathways and butyrate-producing taxa reduces intestinal barrier permeability, limiting biologic drug loss into the gut lumen and correlating with higher trough levels and remission rates [60,89]. This relationship extends to anti-TNF response prediction: Aden et al. reported an 81% reduction in butyrate-synthesis pathway abundance and diminished metabolite exchange within the bacterial community among anti-TNF non-responders, with predicted butyrate and substrate (acetaldehyde, ethanol) levels closely tracking clinical remission [51,60]; Effenberger et al. similarly found 1.7-fold higher butyrate production in responders relative to non-responders. Baseline butyrate-synthesis capacity has also been identified as a significant predictor of azathioprine efficacy, associated with CDAI reduction below 150 and CRP normalization [60,87]. These findings should be interpreted according to the timing of microbiome assessment. A microbial functional profile measured before treatment and associated with a subsequent outcome may serve as a candidate predictive biomarker, whereas reduced butyrate-synthesis capacity observed in non-responders does not, by itself, establish that this feature prospectively predicts treatment failure. Distinguishing these possibilities requires longitudinal studies with clearly defined pretreatment sampling and treatment outcomes.

#### 7.3.3. Tryptophan-Derived Indoles

The AhR-mediated mechanism of indole signaling introduced previously is further substantiated by Lamas et al., who showed—using stool analysis from IBD patients alongside Card9-knockout mouse models—that commensal species including *Lactobacillus reuteri*, *Bacteroides thetaiotaomicron*, *Acinetobacter* and *E. coli* convert dietary tryptophan into indole-3-acetic acid, indole-3-propionic acid, and indole-3-carboxaldehyde, which activate AhR on epithelial cells and group 3 innate lymphoid cells to induce IL-22 production [49]. IBD patients—both CD and UC—show significantly reduced fecal tryptophan and indole-3-acetic acid concentrations and impaired AhR-activating capacity relative to healthy controls (*p* < 0.0001), a deficit linked to the IBD risk variant rs10781499 in CARD9 [90]. Supplementation with indole derivatives or indole-producing bacteria such as *Lactobacillus* restores IL-22 levels, repairs the intestinal barrier and attenuates inflammation [90,91]. AhR activation by tryptophan metabolites has additionally been shown to potentiate the anti-colitic effects of therapeutic compounds such as berberine [56].

Not all studies support a robust predictive role for individual metabolites. In a prospective cohort of 79 patients treated with ustekinumab or vedolizumab Prins et al. found no significant baseline differences in α-diversity (Shannon index) or in concentrations of individual secondary bile acids, including LCA, between responders and non-responders. The only metabolic parameter that distinguished the two groups was the log ratio of primary to secondary bile acids (log PBA/SBA, *p* = 0.035), suggesting that the relative balance between bile acid pools—rather than the absolute abundance of any single metabolite—may carry greater predictive value and cautioning against overreliance on single-metabolite biomarkers [92].

Taken together, these three mechanistic axes—direct immune activation, microbial biotransformation of therapeutic agents, and metabolite-mediated modulation of host immunity and barrier function—indicate that the gut microbiota shapes therapeutic response through converging, only partially independent, pathways. Their relative contribution likely varies by drug class, disease phenotype and individual microbial community structure underscoring the potential of integrated microbiome-metabolome profiling as a tool for treatment stratification in IBD.

#### 7.3.4. L-Ornithine

Beyond anti-TNF agents and small-molecule therapies, emerging evidence suggests that microbiota-derived metabolites may also modulate the response to ustekinumab. Wang et al. identified a link between *Faecalibacterium prausnitzii*, L-ornithine biosynthesis, and higher baseline L-ornithine concentrations in patients with Crohn’s disease. Mechanistic experiments indicated that L-ornithine may enhance sensitivity to ustekinumab by targeting the EGR1–IL-12RB1 axis and modulating downstream TYK2/STAT3 signaling, thereby limiting Th17-cell stabilization and inflammatory responses. Furthermore, the authors observed improved therapeutic efficacy with L-ornithine supplementation in a prospective clinical trial. These findings highlight a potential microbiota-metabolite-host pathway influencing ustekinumab response, although independent validation and further investigation are needed before its clinical implications can be established [82].

## 8. Future Perspecitves

Despite substantial advances in biologic and small-molecule therapy for IBD, treatment failure remains common: approximately 40% of patients derive no clinical benefit from these agents [93], and roughly half fail to achieve remission on monotherapy [94]. The scale of this problem is illustrated by the multicentre J2J study, which examined patients with UC who discontinued their first JAK inhibitor because of lack of efficacy and switched to a second agent. Within this selected population, 47.9% achieved steroid-free remission after switching [41]. These findings highlight the challenges of achieving remission following an inadequate response to an initial JAK inhibitor but should not be interpreted as representing the overall failure rate of JAK inhibitors. This persistent treatment gap underscores the need for reliable predictive biomarkers, and the gut microbiome is increasingly recognized as both an important modulator of therapeutic efficacy and a promising source of such markers. In the phase 3 GARDENIA trial, multivariate models combining baseline microbiome profiles (490 species) with clinical variables showed modest discriminatory performance for predicting remission on infliximab and etrolizumab (AUROC = 0.643), although they outperformed models based on clinical data alone [44]. Separately, AI-driven personalized microbiome modulation has shown promising results in improving anti-TNF response rates and reducing inflammatory markers in a validation cohort [88]. Nevertheless, the modest predictive performance of the GARDENIA model should be considered when assessing its potential clinical utility. Moreover, improved prediction by a multivariable model does not establish the independent predictive contribution of individual microbial features. Before microbiome-based signatures can guide treatment selection, their predictive performance and added value beyond established clinical predictors should be validated in prospective, independent cohorts. Future work should move beyond the largely bacteriocentric focus of studies to date [95] and systematically compare the full gut ecosystem—virome, archaeome, mycobiome and eukaryotic protists (e.g., *Blastocystis* spp.)—between treatment responders and non-responders [43,96]. Realizing this broader view will require expanded reference databases and standardized, cross-kingdom detection protocols [52,95]. Emerging clinical data suggest that mycobiome features may be associated with anti-TNF response, with differences in the abundance of *Candida* and *Saccharomyces* reported between responders and non-responders; however, these findings remain preliminary and require independent validation [97]. Preliminary evidence suggests that gut virome composition may be associated with response to infliximab in pediatric Crohn’s disease; however, these findings are based on a limited cohort and require validation in larger independent studies [98]. Beyond the gut virome, specific viral infections may complicate the assessment of therapeutic response. For example, cytomegalovirus (CMV) colitis can mimic or exacerbate an IBD flare and is associated with poor outcomes in patients with refractory disease; therefore, persistent symptoms during treatment should not automatically be interpreted as evidence of biologic treatment failure without considering concurrent infection [99]. Gut protists may also be relevant to treatment response: experimental studies have shown that *Blastocystis* can induce pro-inflammatory cytokines, including TNF-α, IL-1β and IL-6, although whether these effects contribute to treatment failure in patients with IBD remains unknown [100].

The most promising predictive approach, however, is the integration of microbiome data with host immunological and genetic layers [101,102]. Should such integrated biomarkers be validated, their clinical application could substantially improve pre-treatment therapy selection, sparing patients the burden of empirical drug cycling and its diminishing likelihood of subsequent remission [94], reducing unnecessary exposure to immunosuppressive and biologic agents in predicted non-responders [101] and enabling faster control of inflammation, thereby limiting irreversible structural bowel damage and the need for surgery [102]. These clinical gains could potentially translate into economic benefit: biologic and small-molecule agents constitute a major share of gastroenterology healthcare expenditure [43,102,103] and their ineffective use in non-responders, together with the downstream costs of hospitalization, emergency care, and surgery, represents a substantial potentially avoidable financial burden [88,102]. However, although AI-guided, biomarker-based approaches such as personalized microbiome modulation may improve treatment selection, their cost-effectiveness has not yet been firmly established and requires formal health-economic evaluation.

Prospective validation in well-characterized, independent cohorts, together with standardized sampling and analytical approaches, will therefore be essential to establish the reproducibility and clinical utility of such microbiome-based predictive biomarkers. Integration with host genetic and immunological data may further improve their predictive value and help identify biological mechanisms underlying response and non-response. Nevertheless, as the evidence reviewed here demonstrates, the gut microbiome represents a biologically plausible and increasingly tractable substrate for precision therapeutics in IBD and its systematic, multi-kingdom characterization is likely to be central to closing the persistent gap between the growing number of available advanced therapies and the proportion of patients who ultimately benefit from them.

## Figures and Tables

**Table 1:** Effects of gut microbial taxa, enzymes and metabolites on drug metabolism and therapeutic response in IBD.

Drug/Drug Class	Responsible Bacterial Taxa	Microbial Enzymes/ Metabolite	Mechanism	Functional Consequence	References
Sulfasalazine, olsalazine, balsalazide	*Clostridium* spp., *Eubacterium hadrum*, *Staphylococcus aureus*, *Escherichia coli*, *Klebsiella aerogenes*, *Pseudomonadaceae*, *Bacteroides*, *Lactobacillus acidophilus*, *Bifidobacterium lactis*, *Streptococcus salivarius*	Azoreductases	Azo bond cleavage	Bioactivation: release of 5-ASA	Franzin et al. [37]
Mesalazine	*Citrobacter*, *Klebsiella*, *Serratia*, *Shigella*, *Vibrio*, *Pseudomonas aeruginosa*, *Bacteroides*, *Clostridium*, *Lactobacillus*	N-acetyltransferases (NAT); thiolases; acyl-CoA N-acyltransferases	Acetylation to N-acetyl-5-ASA	Inactivation; association with reduced treatment efficacy	Franzin et al. [37], Delomenie et al. [63], Nanni et al. [62], Mehta et al. [64]
Azathioprine, 6-mercaptopurine, 6-thioguanine	*Escherichia coli* DH5α, *Enterococcus faecalis*, *Bacteroides thetaiotaomicron*, *Bacteroides fragilis*, *Bacteroides vulgatus*, *Proteobacteria*.	hypoxanthine phosphoribosyltransferase (HPRT), HGPRT, inosine-5′-monophosphate dehydrogenase (IMPDH), guanosine monophosphate synthetase (GMPS), glutamine synthetase (GST)	Bioactivation to thioguanine nucleotides (TGN)	Modulation of thiopurine bioactivation and inactivation	Franzin et al. [37], Nanni et al. [62], Oancea et al. [65], Movva et al. [66], Krynetski et al. [67], Liu et al. [68]
	*Pseudomonas syringae*, *Pseudomonas aeruginosa*, *Pseudomonas fluorescens*, *Escherichia coli*	bacterial thiopurine S-methyltransferase (TPMT) orthologs, xanthine oxidase (X0)	Inactivation by methylation/oxydation		
Glucocorticoids	*Gordonibacter pamelaeae*, *Eggerhella lenta*	21-dehydroxylation enzymes	Conversion to progestogens/neurosteroids	Altered glucocorticoid availability and metabolism	Franzin et al. [37], Nanni et al. [62], Yadav et al. [52], Vertzoni et al. [69]
	Other taxa not specified	Glycosidases, sulfatases, reductases	Degradation (prednisolone 3 h; budesonide 7 h; prednisone 96 h)		
Methotrexate	*Escherichia coli*, *Prevotellaceae*, *Anaeroplasmataceae*, *Lactobacillus casei*, *Pseudomonas*, *Clostridium symbiosum*, *Clostridium asparagiforme*,	Carboxypeptidase G2 (CPDG2), glutamate carboxypeptidase II, p-aminobenzoylglutamate hydrolase (PGH)	Hydrolysis of methotrexate and its polyglutamates to less active metabolites, including 4-amino-4-deoxy-N10-methylpteroic acid (APA) and deoxyaminopteroate (DAMPA)	Microbial detoxification and reduced methotrexate activity/toxicity	Franzin et al. [37], Nanni et al. [62], Cho et al. [70], Shin et al. [71]
Ozanimod (S1P modulator)	Gut microbiota (taxa not specified)	Not specified	Reductive metabolism involving oxadiazole ring opening.	Reduced parent-drug availability; <40% of ozanimod remaining after 24 h	Nanni et al. [62], Surapaneni et al. [72], Favaron et al. [73], Collins et al. [74]
Tofacitinib (JAK inhibitor)	Gut microbiota (taxa not specified)	Not specified	No significant microbial degradation detected	No clear evidence of direct microbiota-mediated drug degradation	Bartocci et al. [75], Favaron et al. [73]
Infliximab (anti-TNF)	*Streptococcus pyogenes*	Immunoglobulin-degrading protease (IdeS)	In vitro cleavage of IgG by the IdeS protease produced by *S.pyogenes.*	Potential alteration of monoclonal antibody effector function (In vitro; clinical relevance in IBD remains uncertain)	Estevinho et al. [76], Von Pawel-Rammingen et al. [77], Deveuve et al. [78], Bartocci et al. [75]
Infliximab (anti-TNF)	*Methylobacterium*, *Sphingomonas*, *Staphylococcus*, *Streptococcus*, *Lactococcus*, *Roseburia*, *Faecalibacterium prausnitzii*, *Blautia*, *Faecalibacterium*, *Lachnospira*; *↓ Escherichia*, *Enterococcus*	Short-chain fatty acids (SCFAs), bile salt hydrolases	Association between gut microbiota composition/diversity and anti-TNF treatment response	Potential modulation/prediction of therapeutic response	Estevinho et al. [76], Rajca et al. [79], Wang et al. [80], Ding et al. [81]
Ustekinumab (anti-IL12/123)	*Faecalibacterium prausnitzii*	L-ornithine; acetylornithine aminotransferase	L-ornithine-mediated modulation of the IL-12RB1/TYK2/STAT3 axis	Enhanced ustekinumab treatment sensitivity	Wang et al. [82]
Vedolizumab (anti-integrin)	*Roseburia inulinivorans*, *Phascolarctobacterium faecium*, *Agathobaculum butyriciproducens*, *Clostridium citroniae*, *Anaerostipes hadrus*, *Faecalibacterium prausnitzii*, *Burkholderiales species*	SCFAs, including butyrate; branched-chain amino acid biosynthesis pathways	Microbiota-associated modulation of treatment response	Association with clinical remission and therapeutic drug exposure	Ananthakrishnan et al. [83], Colman et al. [84], Jiang et al. [85]

↓ indicates decreased abundance.

**Table 2:** Microbial and metabolite determinants of therapeutic response in IBD.

Therapy	Microbial/Metabolite Feature	Direction of Association	Evidence Type	Quantitative Data	Reference
Infliximab	*Fusobacterium nucleatum* (NAMPT/NAD^+^ salvage pathway-p38 MAPK))	Higher abundance → non-response	Preclinical (mouse) + clinical association	Pathway inhibition restored infliximab response	Lei et al. [58]
	*Bifidobacterium catenulatum* and taurochenodeoxycholic acid—(TCDCA)	Decrease → non- response	Clinical association + preclinical (FMT)	AUC = 0.805	Liu et al. [57]
	Microbiota diversity, *Faecalibacterium. prausnitzii*	Higher abundance/diversity → response (mucosal healing)	Clinical correlation	No effect size reported	Franzin et al., [37] Seong et al. [86]
Anti-cytokine vs. anti integrin	Functional *bai* operon (*bai^+^*)	Presence → higher remission with anti-cytokine therapy	Clinical (prospective cohort)	OR 3.52 (95% CI 1.30–10.1, *p* = 0.02)	Lee et al. [61]
Anti-TNF	Deoxycholic acid (DCA) vs. conjugated primary bile acids (CPBA)	Higher DCA levels → response Higher CPBA levels → non-response	Clinical, predictive model	AUC = 0.81	Meade et al. [60]
	Butyrate-synthesis pathway abundance	Lower abundance → non-response	Clinical (metagenomic)	81% reduction in non-responders	Wang et al., [51] Meade et al. [60]
	Butyrate production	Higher butyrate producion → response	Clinical	1.7-fold higher in responders	Meade et al., [60] Zhao et al. [87]
	AI-driven personalized microbiome modulation	Intervention → response	Clinical (validation cohort)	Higher response; reduced CRP and calprotectin levels	Goldis et al. [88]
Vedolizumab	Lithocholic acid (LCA), takeda G protein-coupled receptor 5/farnesoid X receptor (TGR5/FXR)	Higher levels → response	Preclinical (mouse) and predictive model	AUC = 0.917	Han et al. [56]
Biologics (general)	Butyrate-producing taxa/SCFA pathways	Higher abundance → response;	Clinical correlation	Reduced drug loss	Meade et al., [60] Malinauskiene et al. [89]
Azathioprine	Baseline butyrate-synthesis capacity	Higher butyrate-synthesis capacity → response	Clinical	CDAI < 150, CRP normalization	Meade et al., [60] Zhao et al. [87]
Infliximab, etrolizumab	Microbiome model (490 species) + clinical vars	Model improves prediction	Clinical, predictive model (GARDENIA); 287 patients with microbiome data; internal cross-validation; no external validation reported	Combined microbiome-clinical model: AUROC = 0.643, AUPRC = 0.401 (baseline AUPRC = 0.265)	Tamburini et al. [44]

## Data Availability

No new data were created or analyzed in this study. Data sharing is not applicable.

## Abbreviations

The following abbreviations are used in this manuscript:

5-ASA	5-aminosalicylic acid
ADA	anti-drug antibodies
AhR	aryl hydrocarbon receptor
AIEC	adherent-invasive *Escherichia coli*
APA	4-amino-4-deoxy-N10-methylpteroic acid
CD	Crohn’s disease
CMV	cytomegalovirus
DCA	deoxycholic acid
FXR	farnesoid X receptor
GBD	Global Burden of Disease
HDAC	histone deacetylase
IBD	inflammatory bowel disease
ILCs	innate lymphoid cells
IL	interleukin
JAK	Janus kinase
LCA	lithocholic acid
NAMPT	nicotinamide phosphoribosyltransferase
NAT	N-acetyltransferases
NR-FMT	fecal microbiota transplantation from non-responders
NLRs	NOD-like receptors
NTBF	non-toxigenic strains of *Bacteroides fragilis*
PSA	polysaccharide A
SCFAs	Short-chain fatty acids
TCDCA	taurochenodeoxycholic acid
TLRs	Toll-like receptors
TGN	thioguanine nucleotides
TPMT	thiopurine methyltransferase
UC	ulcerative colitis
